# Assessment of *vasa vasorum* on coronary plaques in patients with acute coronary syndromes using intravascular ultrasound and iMap analysis: A retrospective cohort study

**DOI:** 10.1097/MD.0000000000034458

**Published:** 2023-07-28

**Authors:** Xi Wu, Gang Ji, Quan Wang, Jing Chen, Xin-Yu Cai, Jing Song, Yan Yan, He Huang

**Affiliations:** a Department of Cardiology, Xiangtan Central Hospital, Xiangtan, Hunan, China.

**Keywords:** intravascular ultrasound, plaque vulnerability, vasa vasorum

## Abstract

Studies have revealed that *vasa vasorum* (VV) neovascularization is vital for the progression and vulnerability of coronary atherosclerotic plaques. The correlation between VV, plaque constituents, and the no-reflow phenomenon (NRP) in percutaneous coronary intervention (PCI) remains elusive. We explored plaque constituents in iMap-intravascular ultrasound (iMap-IVUS) and NRP during PCI for VV lesions. We studied 166 coronary lesions in 166 patients with acute coronary syndromes (ACS) (118 lesions with VV) undergoing pre-intervention intravascular ultrasound (IVUS). We evaluated the diversity in plaque morphological status and post-PCI results based on the presence or absence of VV. The lesions with VV group had significantly higher high-sensitivity C-reactive protein (hs-CRP) levels than the lesions without VV group (8.41 ± 4.98 vs 4.19 ± 3.69 mg/L, *P <* .001). The frequency of after-stent deployment thrombolysis in myocardial infarction (TIMI) flow grades 0, 1, and 2 was remarkably greater in lesions with VV than in those without VV (22.9% vs 10.4%, *P* < .001). Plaques at the minimum lumen, necrotic core (1.26 ± 0.64 vs 0.92 ± 0.61 mm^2^, *P* < .001; 20.95 ± 7.19 vs 13.34% ± 6.54%, *P* < .001), and fibrous areas (4.23 ± 1.32 vs 3.92 ± 1.01 mm^2^, *P* = .006; 61.01 ± 9.41 vs 56.92% ± 11.42%, *P =* .001) were considerably larger in the lesions with VV than in those without VV. In addition, densely calcified plaques (0.41 ± 0.26 vs 0.81 ± 0.59 mm^2^, *P* < .001; 3.63 ± 2.19 vs 7.18% ± 2.01%, *P* < .001) were considerably smaller in the lesions with VV than in those without VV. Multivariate analyses revealed that VV and plaque volume were independent predictors of NRP after stent deployment (odds ratio [OR]: 5.13, 95% confidence interval [CI]: 1.19–15.32, *P* = .002; OR: 4.79, 95% CI: 1.08–9.01, *P* = .005). Lesions with VV exhibited considerable plaque vulnerability in patients with ACS, and they displayed more NRP during PCI. VV and plaque volume were independent predictors of NRP after stent deployment.

## 1. Introduction

The coronary *vasa vasorum* (VV) is a net of microvessels in the coronary artery walls that is often identified in patients with acute myocardial infarction, lesions with thin-capped fibroatheroma (TCFA), or plaque disruption.^[1–4]^ Hence, VV is considered a morphological index for vulnerable plaques and plaque progression. VV is identified via computed tomography (CT) and optical coherence tomography (OCT) as nets of microvessels with 50 to 150 μm diameter.^[5,6]^ Nevertheless, CT has radiation-related issues, making identifying plaque features using CT challenging. Furthermore, the penetration depth of OCT is unsatisfactory; hence, identifying the entire VV might be challenging in vessels with larger diameters. Intravascular ultrasound (IVUS) can penetrate 8 to 10 mm deep; thus, it is more appropriate for determining VV in deeper regions. Grayscale IVUS is utilized to determine the morphometrical measurement of coronary arteries, identifying VV in vivo as a tubular, low-echo architecture at the outer side of a plaque.^[7]^ Nevertheless, traditional IVUS has remarkable deficiencies in evaluating plaque constituents.^[8]^ iMap-intravascular ultrasound (iMAP-IVUS) can analyze radiofrequency signals obtained using IVUS and categorize plaques into fibrosis, lipidic, dense calcium, and necrotic core.^[9]^ Furthermore, plaque constituents are related to coronary events.^[10]^ Nevertheless, the influence of VV on local lesion characteristics and patient clinical features—visualized using IVUS—has not been investigated.

Therefore, this study aimed to reveal the association between VV architecture and plaque features via iMAP-IVUS in patients with acute coronary syndrome (ACS).

## 2. Materials & methods

### 2.1. Research population

**Figure 1.**
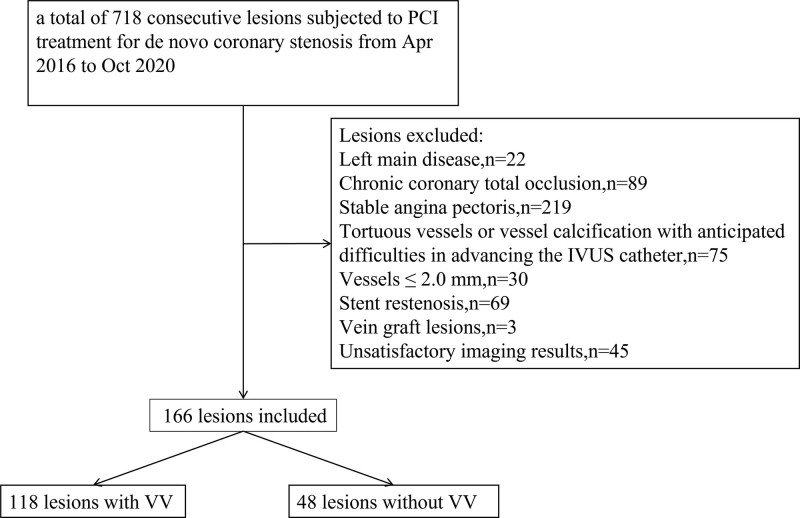
The study flow chart. IVUS = intravascular ultrasound, PCI = percutaneous coronary intervention, VV = *vasa vasorum.*

This was a retrospective, single-center study of 166 consecutive patients with ACS with 166 lesions who underwent percutaneous coronary intervention (PCI) treatment for de novo coronary stenosis at Xiangtan Central Hospital between April 2016 and October 2020 (Fig. 1). Based on the appearance of VV in the culprit lesion, the study participants were split into 2 groups. Satisfactory imaging results were acquired via IVUS prior to PCI.

The exclusion criteria were as follows: left main disease; chronic coronary total occlusion; stable angina pectoris (SAP); tortuous vessels or vessel calcification with anticipated difficulties in advancing the IVUS catheter; vessels of ≤ 2.0 mm, stent restenosis; vein graft lesions; and unsatisfactory imaging results. This study was conducted in accordance with the Declaration of Helsinki and approved by the Ethical Board of Xiangtan Central Hospital. We acquired written informed consent from each patient before the study: ACS involving unsteady angina pectoris, ST-segment elevation myocardial infarction (STEMI), and non-STEMI. Unstable angina pectoris was identified by chest pain within the preceding 72 hours with or without ST-T segment variations or positive heart chemical biomarkers. STEMI was identified by persistent chest pain (0.5 hours), a new ST-segment elevation of 2 mm on ≥2 consecutive electrocardiography leads, and a creatine kinase-MB level > 3 times the standard.^[11]^ Non-STEMI was identified by chest pain and a positive heart chemical biomarker without new ST-segment elevation.^[12]^ The culprit vessel was confirmed based on clinical, electrical cardiogram, and angiography results.

### 2.2. PCI and IVUS procedure

All patients received aspirin (100 mg/day), clopidogrel (75 mg/day), or ticagrelor (180 mg/day) before the PCI. Heparin (80 U/kg) was intravenously administered at the start of catheterization. Pre-intervention angiography was completed posterior to the intra-coronary administration of 0.1 to 0.2 mg nitroglycerin. When substantial thrombi were observed, IVUS was completed posterior to thrombus aspiration. This procedure was completed before dilatation using a suitable balloon and stent deployment. The thrombus is considered a discrete intraluminal-filling flaw.^[13]^ Coronary flow was evaluated based on the thrombolysis in myocardial infarction (TIMI) flow grade.^[14]^ The angiographic no-reflow phenomenon (NRP) was defined as a reduction of ≥1 grade in TIMI flow after stent deployment or an eventual TIMI flow grade of 0 and 1 or 2 without proof of thrombi, spasm, or dissection. The main operator determined the use of GPIIb/IIIa inhibitors, intracoronary nitroglycerin (100–200 μg bolus), intracoronary sodium nitroprusside (100–200 μg bolus), intracoronary adenosine (40–200 μg bolus), and intracoronary nicorandil (1–2 mg bolus). After the patient was administered 0.1 to 0.2 mg nitroglycerin inside the coronary artery, a 40-MHz rotational IVUS catheter (Opticross, Boston Scientific, America) was pulled back at 0.5 mm/s from the distal spot of the culprit morbid change to the aorto-ostial junction.

### 2.3. Grayscale IVUS and iMAP-IVUS analyses

**Figure 2.**
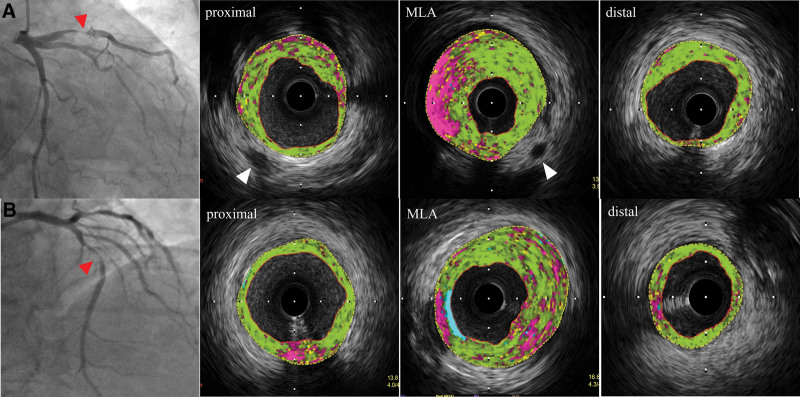
Representative angiographic and iMAP-IVUS images of coronary plaques (red arrowhead) with or without VV (A, B). (A) Plaque with VV. Tubular, low echoic structures exterior to the media, indicating VV (white arrowhead) at the 5 and 7 o’clock positions. (B) Plaque without VV. Plaque components were classified as fibrotic (light green), lipidic (yellow), necrotic (pink), or calcified (light blue). MLA = minimum lumen area, VV = *vasa vasorum.*

The IVUS images of each patient were documented and preserved on a DVD-ROM. Two researchers blinded to the patient information or angiogram result completed the offline analyses. A consensus was reached via discussion during disagreements regarding the analytical results. Quantitation and qualitative IVUS analyses were completed according to the American College of Cardiology Clinical Expert Consensus Document on Standards for Acquisition, Measurement, and Reporting of Intravascular Ultrasound Studies.^[15]^ Through computerized planimetric analyses (Echo Plaque, Index System), culprit lesions and referential segments were evaluated. The minimal luminal area (MLA) was the site with the least cross-sectional area within the lumen. The referential spots comprised the most normal cross-sectional areas within 10 mm proximal and distal to the lesion. IVUS measurement of the external elastic membrane (EEM), lumen, and plaque plus media (P + M = EEM—lumen) at the MLA spot in and at a reference segment near the culprit lesion. The (P + M) percentage was computed as the P + M area divided by the EEM area at the MLA spot. The remodeling index was calculated as the EEM area at the MLA spot divided by the average reference EEM area. Positive remodeling was defined as a remodeling index of > 1.05. The VV was a small (<1 mm) tubular or vesicular, low-echo architecture exterior to the media (≤1 mm) (Fig. 2).^[7]^ Attenuated plaques were ultrasonic attenuation of >90° of the plaque.^[16]^ Volumetric analysis was performed on the 10 mm vessels, and using Simpson rule, the segment with the smallest lumen area was considered the center. iMAP-IVUS analysis was completed using the iMap IVUS QIvus 2.1 system software (Medis Medical Imaging System, the Netherlands).^[9]^ Vascular and luminal borders were traced via automatic edge identification and artificially calibrated. Plaque constituents were categorized as fibrosis (light green), lipid (yellow), necrosis (pink), or calcification (light blue) and were depicted as absolute areas (mm^2^) and percentages (%) of the overall plaque area (Fig. 2). iMAP-derived TCFA was at least 1 frame with > 30° of necrotic core abutting the lumen.^[17]^

### 2.4. Clinical follow-up

Physicians collected information during new hospitalizations, telephone calls, or clinic visits after PCI. The primary endpoints for all patient outcome comparisons were major adverse cardiac and cerebrovascular events (MACCE), a composite of all-cause death, cardiac death, recurrent myocardial infarction, target lesion revascularization, re-hospitalization, heart failure, and stroke.

### 2.5. Statistics

Statistical analyses were performed using IBM SPSS Statistics 22.0 (America). Continuous data are presented as the mean ± SD. The classification data are presented as proportions (%). Continuous variables were compared using the unpaired Student *t* test. Categorical variables were compared using the chi-square or Fisher exact test. Independent predictive factors for NRP posterior to stent implantation were determined via multivariate logistic regression analyses and presented as odds ratios (OR) and 95% confidence intervals (CIs). We introduced variates with *P* < .1 in the univariate and multivariate models. After forming the stepdown logistic regression, the least significant variable was dropped at each step until only the variable with *P* < .05 remained. Statistical significance was set at *P* < .05.

## 3. Results

### 3.1. Baseline clinical features

Overall, 166 lesions were included, of which 118 (71.1%) had VV. Table 1 presents the baseline clinical features of the patients. No evident differences were observed in age and sex between these groups. Coronary artery disease risk factors such as hypertension, diabetes, smoking, and family history did not differ between groups. Moreover, no remarkable differences were identified in the prior myocardial infarct, left ventricular ejection fraction, body mass index, target coronary artery, triglycerides, total cholesterol, low-density lipoprotein-C, and high-density lipoprotein-C. Lesions with VV had significantly higher high-sensitivity C-reactive protein (hs-CRP) levels (8.41 ± 4.98 vs 4.19 ± 3.69, *P* < .001) than those without VV.

**Table 1:** Baseline clinical characteristics.

Variables	Lesions with VV (n = 118)	Lesions without VV (n = 48)	*P* value
Age (yr)	62.71 ± 6.36	61.55 ± 6.08	.401
Male, n(%)	81 (68.6)	34 (70.8)	.587
Hypertension, n(%)	65 (55.1)	29 (60.4)	.292
Diabetes, n(%)	42 (35.6)	14 (29.2)	.173
Current smokers, n(%)	36 (30.5)	14 (27.1)	.604
Prior myocardial infarction, n(%)	27 (22.9)	10 (20.8)	.822
Family history of CAD, n(%)	70 (59.3)	22 (45.8)	.115
History of dyslipidemia, n(%)	45 (38.1)	19 (39.6)	.924
LVEF (%)	62.70 ± 7.69	63.74 ± 8.07	.212
BMI, kg/m^2^	24.82 ± 3.61	23.80 ± 4.33	.197
Target coronary artery, n(%)			.707
LAD	53 (44.9)	23 (47.9)	
LCX	27 (22.9)	9 (18.8)	
RCA	38 (32.2)	16 (33.3)	
Triglycerides (mmol/L)	1.46 ± 0.93	1.58 ± 1.17	.361
Total cholesterol (mmol/L)	4.22 ± 1.12	3.87 ± 1.21	.148
HDL-C (mmol/L)	1.11 ± 0.41	1.28 ± 0.62	.074
LDL-C (mmol/L)	2.96 ± 0.82	2.84 ± 0.92	.513
hs-CRP (mg/L)	8.41 ± 4.98	4.19 ± 3.69	<.001
Baseline TNT (ng/mL)	3.39 ± 2.14	3.07 ± 1.89	.102
Baseline CK-MB (IU/L)	91.51 ± 57.48	88.99 ± 69.11	.403
Medical therapies before admission, n(%)			
Aspirin	118 (100)	48 (100)	>.999
P2Y12 inhibitor	115 (97.5)	46 (95.8)	.615
AECI or ARB	81 (68.6)	34 (70.8)	.873
Beta-blocker	35 (29.7)	11 (22.9)	.233
Statin	93 (78.8)	34 (70.8)	.378
Nitrate	83 (70.3)	33 (68.8)	.793
Hemoglobin A1c (%)	7.51 ± 2.80	7.39 ± 2.41	.405
CKD, n(%)	10 (8.5)	3 (6.3)	.576

Values are mean ± SD, n (%), n/N (%).

ACEI = angiotensin-converting enzyme inhibitors, ACS = acute coronary syndrome, ARB = angiotensin receptor blockers, BMI = body mass index, CAD = coronary artery disease, CKD = chronic kidney disease, CK-MB = creatine kinase MB, HDL-C = high-density lipoprotein cholesterol, hs-CRP = high sensitivity C-reactive protein, LAD = left anterior descending, LCX = left circumflex, LDL-C = low-density lipoprotein cholesterol, LVEF = left ventricular ejection fraction, RCA = right coronary artery, SAP = stable angina pectoris, TNT = troponin-T, VV = *vasa vasorum*.

### 3.2. Angiography and procedure characteristics

Table 2 presents the angiographic and procedural characteristics. The lesions with VV had remarkably more thrombi prior to PCI than those without VV (56.8% vs 27.1%, *P* < .001).Maximal stent diameters were remarkably greater in the lesions with VV than in those without VV (3.42 ± 0.52 mm vs 3.21 ± 0.48 mm, *P* = .013). The frequency of initial and after stent deployment TIMI flow grade 0, 1, and 2 was remarkably higher in lesions with VV than in lesions without VV (18.6% vs 6.3%, *P* = .001; 22.9% vs 10.4%, *P* < .001, respectively).

**Table 2:** Angiographic and procedure characteristics.

Variables	Lesions with VV (n = 118)	Lesions without VV (n = 48)	*P* value
Thrombectomy, n(%)	35 (29.7)	8 (16.7)	<.001
Thrombus before PCI, n(%)	67 (56.8)	13 (27.1)	<.001
Before dilatation, n(%)	169 (100)	216 (100)	>.999
Maximal stent diameter (mm)	3.42 ± 0.52	3.21 ± 0.48	.013
Stent length (mm)	28.91 ± 5.56	28.79 ± 5.71	.489
Maximal stent pressure (atm)	8.61 ± 2.84	8.44 ± 2.51	.672
Maximum balloon pressure (atm)	15.39 ± 2.37	15.17 ± 2.43	.611
Initial TIMI flow grade 0/1/2, n(%)	22 (18.6)	3 (6.3)	.001
Final TIMI flow grade 0/1/2, n(%)	2 (1.7)	0 (0)	.305
Before stenting TIMI flow grade 0/1/2, n(%)	4 (3.4)	1 (2.1)	.639
After stenting TIMI flow grade 0/1/2, n(%)	27 (22.9)	5 (10.4)	<.001

Values are mean ± SD, n (%), n/N (%).

PCI = percutaneous coronary intervention, TIMI = thrombolysis in myocardial infarction, VV = *vasa vasorum*.

### 3.3. Grayscale IVUS and iMAP-IVUS findings

At the MLA, the P + M area was considerably larger, whereas the lumen area was considerably smaller in the lesion with VV compared to the lesion without VV (10.31 ± 2.31 mm^2^ vs 9.07 ± 2.29 mm^2^, *P* < .001; 1.99 ± 1.31 mm^2^ vs 2.52 ± 1.46 mm^2^, *P* < .001, respectively). The frequency of attenuated plaques was higher in lesions with VV than in those without VV (32.2% vs 10.4%, *P* < .001). In the iMAP-IVUS analysis, plaques at MLA, fibrous areas, and necrotic core areas were considerably larger in lesions with VV than in those without VV (4.23 ± 1.32 mm^2^ vs 3.92 ± 1.01 mm^2^, *P* = .006; 61.01 ± 9.41% vs 56.92 ± 11.42%, *P* = .001; 1.26 ± 0.64 mm^2^ vs 0.92 ± 0.61 mm^2^, *P* < .001; 20.95 ± 7.19% vs 13.34 ± 6.54%, *P* < .001, respectively). In addition, densely calcified areas were considerably smaller in lesions with VV than in those without VV (0.41 ± 0.26 mm^2^ vs 0.81 ± 0.59 mm^2^, *P* < .001; 3.63 ± 2.19% vs 7.18 ± 2.01%, *P* < .001, respectively). The frequency of iMAP-derived TCFAs and attenuated plaques was considerably higher in lesions with VV than in those without VV (29.7% vs 6.2%, *P* < .001; 32.2% vs 10.4%, *P* < .001, respectively). The plaque volume was considerably larger in lesions with VV than in those without VV (90.32 ± 12.54 mm^3^ vs 77.31 ± 10.49 mm^3^, *P* < .001). Additionally, no remarkable diversity was observed in grayscale IVUS and iMAP-IVUS variables at the proximal or distal referential spots between lesions with and without VV (Table 3).

**Table 3:** Greyscale IVUS and iMAP-IVUS findings.

Variables	Lesions with VV (n = 118)	Lesions without VV (n = 48)	*P* value
IVUS Lesion length (mm)	25.21 ± 4.31	24.84 ± 5.12	.401
Proximal reference			
EEM area (mm^2^)	14.19 ± 0.82	14.34 ± 0.71	.192
P + M area (mm^2^)	7.43 ± 0.43	7.39 ± 0.41	.295
Lumen area (mm^2^)	7.41 ± 0.67	7.38 ± 0.51	.301
%P + M area (%)	56.91 ± 4.97	55.36 ± 5.05	.206
Fibrous area (mm^2^)	2.39 ± 0.71	2.31 ± 0.66	.112
Lipidic area (mm^2^)	0.94 ± 0.71	0.91 ± 0.66	.339
Densely calcified area (mm^2^)	0.41 ± 0.43	0.49 ± 0.37	.214
Necrotic core area (mm^2^)	0.52 ± 0.48	0.53 ± 0.29	.408
Fibrous area (%)	56.49 ± 12.91	54.91 ± 13.02	.192
Lipidic area (%)	22.81 ± 9.42	21.39 ± 10.08	.518
Densely calcified area (%)	10.31 ± 7.91	11.39 ± 7.01	.202
Necrotic core area (%)	12.89 ± 7.19	10.13 ± 8.04	.101
Distal reference			
EEM area (mm^2^)	9.54 ± 1.43	9.31 ± 1.81	.243
P + M area (mm^2^)	5.61 ± 2.41	5.72 ± 2.32	.082
Lumen area (mm^2^)	4.21 ± 2.66	4.37 ± 2.09	.135
%P + M area (%)	43.91 ± 15.21	42.41 ± 14.91	.321
Fibrous area (mm^2^)	1.09 ± 0.71	1.16 ± 0.61	.319
Lipidic area (mm^2^)	0.59 ± 0.31	0.52 ± 0.41	.291
Densely calcified area (mm^2^)	0.45 ± 0.31	0.43 ± 0.92	.409
Necrotic core area (mm^2^)	0.35 ± 0.18	0.32 ± 0.21	.512
Fibrous area (%)	45.32 ± 13.11	44.91 ± 14.09	.783
Lipidic area (%)	25.94 ± 10.32	24.69 ± 11.14	.079
Densely calcified area (%)	15.13 ± 10.21	14.41 ± 11.29	.181
Necrotic core area (%)	14.36 ± 9.12	13.91 ± 9.21	.632
Minimum lumen area			
EEM area (mm^2^)	12.32 ± 2.71	12.22 ± 2.42	.413
P + M area (mm^2^)	10.31 ± 2.31	9.07 ± 2.29	<.001
Lumen area (mm^2^)	1.99 ± 1.31	2.52 ± 1.46	<.001
%P + M area (%)	85.32 ± 10.15	80.42 ± 11.19	<.001
Fibrous area (mm^2^)	4.23 ± 1.32	3.92 ± 1.01	.006
Lipidic area (mm^2^)	1.22 ± 0.25	1.22 ± 0.31	.872
Densely calcified area (mm^2^)	0.41 ± 0.26	0.81 ± 0.59	<.001
Necrotic core area (mm^2^)	1.26 ± 0.64	0.92 ± 0.61	<.001
Fibrous area (%)	61.01 ± 9.41	56.92 ± 11.42	.001
Lipidic area (%)	16.43 ± 6.12	17.21 ± 5.59	.554
Densely calcified area (%)	3.63 ± 2.19	7.18 ± 2.01	<.001
Necrotic core area (%)	20.95 ± 7.19	13.34 ± 6.54	<.001
Remodeling index	1.09 ± 0.11	1.01 ± 0.14	.052
iMAP-derived TCFAs at MLA, n(%)	35 (29.7)	3 (6.2)	<.001
Attenuated plaques at MLA, n(%)	38 (32.2)	5 (10.4)	<.001
Plaque volume (mm^3^)	90.32 ± 12.54	77.31 ± 10.49	<.001
Positive remodeling, n(%)	30 (25.4)	9 (18.8)	.063

Values are mean ± SD, n (%), n/N (%).

EEM = external elastic membrane, IVUS = indicates intravascular ultrasound, MLA = minimal luminal area, PCI = indicates percutaneous coronary intervention, P + M = plaque plus media, TCFA = thin cap fibroatheroma, VV = *vasa vasorum*.

### 3.4. Predictors of no-reflow after stent deployment

**Figure 3.**
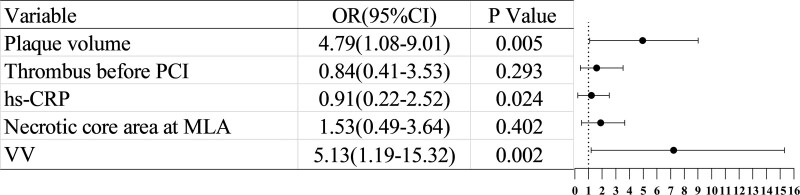
Independent predictors of no-reflow after stent implantation via multivariate analyses. PCI = percutaneous coronary intervention; CI = confidence interval, hs-CRP = high-sensitivity C-reactive protein, MLA = minimum lumen area, OR = odds ratio, VV = vasa vasorum.

Multivariate analyses revealed that VV and plaque volume were independent predictors of NRP after stent deployment (OR: 5.13, 95% CI: 1.19–15.32, *P* = .002; OR: 4.79, 95% CI: 1.08–9.01, *P* = .005) (Fig. 3).

### 3.5. Clinical follow-up outcomes

The median follow-up period was 36 months (14–79 months). Follow-up was accomplished for 90% of the patients. In patients with VV lesions with VV, 1 patient with NRP developed in-hospital cardiac death after stenting. Among patients having lesions without VV, none developed in-hospital MACCEs. During the follow-up, 9 MACCE events occurred. Among patients having lesions with VV, 1 cardiac death, three recurrent myocardial infarctions, 2 target lesion revascularizations, and 3 heart failures occurred. Regarding patients having lesions without VV, 1 recurrent myocardial infarction, 1 target lesion revascularization, and 1 heart failure occurred.

## 4. Discussion

This study explored the association between IVUS-identified VV and plaque constituents and investigated NRP during PCI. This study mainly revealed the following: Lesions with VV had higher hs-CRP levels and higher frequencies of thrombi prior to PCI; iMAP-IVUS analysis uncovered that lesions with VV had greater necrotic core and fibrous areas, lower densely calcified areas, larger plaque volumes, and more iMAP-derived TCFAs than lesions without VV at the MLA; NRP during PCI was often identified in lesions with VV; VV and plaque volume were independent predictors of NRP after stent deployment.

### 4.1. Relationship between VV and clinical characteristics

Herein, VV lesions were more frequently identified in patients with ACS and displayed more thrombi. Previous animal and pathological studies have revealed that VV neovascularization might be associated with plaque progression and vulnerability.^[18,19]^ Plaque vulnerability to neovascularization is due to intra-plaque bleeding from non-mature and leaky neovascularization, causing lumen narrowing or embolism.^[20]^ Furthermore, VV is often identified in patients with ACS and lesions with plaque rupture.^[1,3]^ These reports corroborated our research outcomes, where more IVUS-identified VV were detected in patients with ACS and thrombotic lesions. Kume et al observed that VV incidence at the culprit lesions was similar between patients with ACS and those with SAP (97% vs 93%, *P* = .216); Nevertheless, the maximal VV quantity was considerably higher in patients with ACS than in those with SAP (at the culprit lesion: 2.8 ± 1.3 vs 1.8 ± 1.0, *P <* .001, proximal referential site: 1.9 ± 1.1 vs 1.3 ± 0.9, *P* = .003, and distal referential site: 1.7 ± 1.1 vs 1.1 ± 1.1, *P* = .003, respectively).^[21]^ Moreover, low-echo architectures exterior to the media in grayscale IVUS are coherent with VV.^[7]^ Amano et al studied 106 lesions subjected to Virtual histology-IVUS prior to PCI, and discovered that lesions with low-echo architectures on grayscale IVUS displayed great plaque vulnerability and were more often identified in patients with ACS (53% vs 20%, *P* < .001).^[22]^ Hyperlipidemia and hypertension coexistence has been suggested to impair VV formation, resulting in insufficient compensation for hypoxia in thickened media.^[23]^ In this study, coronary artery disease risk factors did not differ between the 2 groups, and lesions with VV had considerably higher hs-CRP levels than those without VV. Pathologic studies have suggested that plaque rupture often occurs where the macrophage foam cell infiltration is most severe. The macrophage foam cells at the plaque rupture site activate inflammatory reactions, elevating hs-CRP levels.^[24]^ Our results are consistent with data from previous studies. An OCT study reported that rupture-related VV correlated with hs-CRP levels.^[6]^

### 4.2. Relationship between VV and plaque compositions

In this study, plaques in the MLA, fibrous and necrotic core areas were considerably larger in lesions with VV. The densely calcified areas were considerably smaller in the lesions with VV. This finding is consistent with the outcomes of previous in vivo and animal studies.^[25,26]^ Moreover, fibrous plaques are in the initiation and progression phases, whereas fibrocalcific plaques are in the static phase.^[27]^ Delivering nutrition and oxygen from the adventitia to the vascular wall is VV main function; therefore, such a positive association is derived from the substantial requirement of nutrition and oxygen during the plaque progression phase.^[28]^ In addition, a pathological study revealed that the necrotic core originated from erythrocyte membranes.^[18]^ Disruption of the arborization architecture of intraplaque new vessels and/or direct supply through the intraplaque new vessels could be the primary source of erythrocyte membranes for vulnerable plaque.

### 4.3. Relationship between VV and TCFAs

A pathological study revealed that lesions with VV display high levels of TCFAs.^[6]^ This report is consistent with the outcomes of this study, in which the iMAP-derived TCFA frequency was higher in VV lesions. Moreover, patients with ACS or lesions with TCFAs displayed a higher frequency of VV; hence, VV might also have vulnerable plaques in lesions with ACS and TCFAs.^[22]^ A previous study revealed that echo-attenuated plaques in patients with ACS display a greater frequency of lipid-rich plaques or TCFAs than those in patients with SAP.^[29]^ OCT studies have revealed that intra-plaque microchannels have more TCFAs, thinner fibrous caps, and hs-CRP content; hence, intra-plaque microchannels have highly vulnerable plaques.^[6]^ This outcome is consistent with our finding that VV displayed plaque vulnerability in lesions of patients with ACS and TCFAs.

### 4.4. Relationship between VV and NRP in PCI

In this study, lesions with VV displayed more NRP during PCI, and VV was an independent predictor of NRP after stent deployment. The etiopathogenesis of NRP is multifactorial, with 1 factor being the mechanical fragmentation of the vulnerable plaque during PCI, causing distal embolization of thrombi, substantial lipidic content, and plaque debris disturbing the coronary microcirculation with an element of elevated local thrombogenicity.^[30]^ The necrotic core constituent of the virtual histology comprises vulnerable tissue, such as cholesterol crystals, lipidic deposition with foam cells, microcalcification, and intramural hemorrhage that undergo mechanical fragmentation during PCI and embolize to the distal coronary microcirculation, facilitating NRP.^[10,31,32]^ TCFA, characterized by fibrosis tissue thickness of <65 μm with a potential necrosis core, is the precursor lesion for plaque rupture.^[33]^ In this study, lesions with VV displayed substantial TCFAs and larger necrotic core content. Distal embolization was probably caused by iatrogenesis of intra-plaque hemorrhage and rupture of TCFAs by balloon dilatation. Microcirculation damage and aberrant tissue perfusion are common after primary PCI, even with TIMI grade 3 flow (normal epicardium flow).^[34]^ Hence, regardless of the causal link of the NRP or its definition, instantaneous angiographic no-reflow seems to signify poor prognoses, as it indicates the existence of tissular malperfusion. Normal, instantaneous, and continuous no-reflow probably pertain to a spectrum of myocardial reperfusion denoting graded microvascular and tissue damage levels, especially during primary PCI in ACS.^[35]^ The severity and duration of instantaneous and continuous no-reflow (as an angiographic surrogate for ischemia-reperfusion damage or distal embolization) can clinically identify the severity and extent of myocyte damage and the final results.^[36]^

## 5. Limitations

First, this was a single-center retrospective study; hence, selection bias might have occurred, possibly excluding lesions that are challenging to identify via IVUS. Therefore, the study outcomes should be confirmed by prospective studies. Second, IVUS did not identify VV in coronary plaques—intraplaque microchannels on OCT. Attenuated and remarkably calcified plaques might affect VV visualization owing to shadowing. Neighboring architectures, such as small branches, veins, and small coronary fistulas, might influence VV identification. In these morbid changes, IVUS may underestimate the frequency and quantity of VV. Third, we could not distinguish atherosclerotic plaques from thrombi, as iMAP-IVUS could not identify thrombi, possibly affecting TCFA determination. Additionally, we did not histopathologically verify plaque vulnerability constituents. This study findings have not been validated by pathological examination. Thus, further research on substantial lesions is required to analyze the coherence between IVUS discoveries and pathology. Therefore, future research should focus on various lesions to examine the consistency between IVUS and pathology results. Few patients who could undergo IVUS tests participated in the current investigation, which was a retrospective, single piece of evidence. Therefore, additional large-scale studies are required.

## 6. Conclusions

VV lesions had highly vulnerable plaques in patients with ACS. These lesions had larger necrotic core and fibrous areas, smaller densely calcified areas, larger plaque volumes, and a higher frequency of iMAP-derived TCFAs. Additionally, they displayed more NRP than lesions without VV during PCI. Multivariate analyses revealed that VV and plaque volume were independent predictors of no-reflow after stent deployment.

## Acknowledgments

We are grateful to Bo Chen for their secretarial assistance.

## Author contributions

**Conceptualization:** Xi Wu, He Huang.

**Data curation:** Xin-Yu Cai, Jing Song.

**Formal analysis:** Quan Wang, Yan Yan.

**Investigation:** Gang Ji.

**Methodology:** Jing Song, Yan Yan.

**Resources:** Jing Chen.

**Software:** Jing Song, Yan Yan.

**Supervision:** Yan Yan.

**Writing – original draft:** Xi Wu.

**Writing – review & editing:** Jing Chen.
